# The relationships between the gut microbiota and its metabolites with thyroid diseases

**DOI:** 10.3389/fendo.2022.943408

**Published:** 2022-08-18

**Authors:** Wen Jiang, Ganghua Lu, Dingwei Gao, Zhongwei Lv, Dan Li

**Affiliations:** ^1^ Department of Nuclear Medicine, Shanghai Tenth People’s Hospital, Tongji University School of Medicine, Shanghai, China; ^2^ Clinical Nuclear Medicine Center, Tongji University School of Medicine, Shanghai, China; ^3^ Institute of Nuclear Medicine, Tongji University School of Medicine, Shanghai, China; ^4^ Department of Nuclear Medicine, Sun Yat-sen Memorial Hospital, Sun Yat-sen University, Guangzhou, China

**Keywords:** thyroid, gut microbiota, thyroid cancer, Hashimoto’s thyroiditis, Graves’ disease, hypothyroidism

## Abstract

Emerging studies have provided a preliminary understanding of the thyroid-gut axis, indicating that intestinal microbiota and its metabolites may act directly or indirectly on the thyroid by influencing intestinal microelements uptake, iodothyronine conversion and storage, and immune regulation, providing new insights into the pathogenesis of thyroid disorders and clinical management strategies. However, the research on gut microbiota and thyroid has only presented the tip of the iceberg. More robust clinical data and basic experiments are still required to elucidate the specific relationships and mechanisms in the future. Here we will characterize the associations between the microbiota and thyroid diseases to evaluate their potential implications in the pathophysiology and open up scientific avenues for future precision studies of the thyroid-gut axis.

## Introduction

Several microorganisms colonize many places in the human body including the oral cavity, respiratory tract, skin, gastrointestinal tract, and genitourinary tract, and form a complex micro-ecosystem within the human body, among which the most complex is found in the intestinal tract. Previous studies demonstrated that the human intestinal microbiota is mainly composed of bacteria, more than 90% of which comprises *Firmicutes*, *Bacteroides*, *Actinomycetes*, and *Proteobacteria*. In addition, other microbes like viruses, fungi, parasites, archaea, etc., are part of the intestinal microbiome composition (1, 2). The 16S rRNA gene and metagenome sequencing studies revealed that there are thousands of different bacterial populations in an adult intestine, encoding approximately 3.3 million genes, which is approximately 100 times the number of genes in the human body (3, 4). The intestinal microbiota and its metabolites are diverse and perform various essential regulating functions, like host nutrient metabolism, xenobiotic and drug metabolism, maintenance of structural integrity of the gut mucosal barrier, immunomodulation, and protection against pathogens (5, 6). The composition and abundance of the intestinal microbiota are dynamic and can be influenced by both genetic and environmental factors. In the event of disturbance of intestinal microbiota’s ecological balance, the host’s normal physiological functions may become compromised, leading to the development of related diseases.

The interactions of microbiota with humans have become one of the most exciting fields, drawing more attention of scientists researching the influence of gut microbiota on various diseases. Studies have demonstrated a strong association between intestinal microbiota and diseases like gastrointestinal diseases (e.g., gastroenteropancreatic neuroendocrine neoplasms, inflammatory bowel disease, and colon cancer), psychiatric diseases (e.g., Alzheimer’s disease, multiple sclerosis, and Parkinson’s disease), respiratory diseases (e.g., bronchial asthma, chronic obstructive pulmonary disease, and infectious lung diseases), cardiovascular diseases (e.g., atherosclerosis), metabolic diseases (e.g., obesity and type 2 diabetes), immune diseases (e.g., rheumatoid arthritis and ankylosing spondylitis), and so on (7–10).

**Figure 1:**
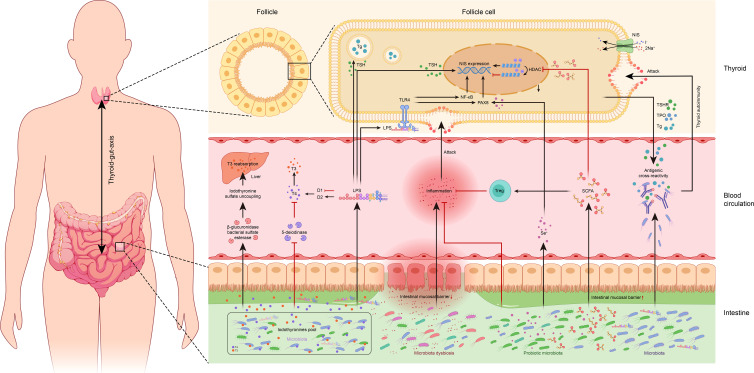
Overview of potential associations between the gut microbiota and thyroid. TSH, thyroid-stimulating hormone; Tg, thyroglobulin; TSHR, thyroid-stimulating hormone receptor; TPO, thyroid peroxidase; NIS, sodium/iodide symporter; HDAC, histone deacetylase; TLR-4, toll-like receptor 4; LPS, lipopolysaccharide; NF-κB, nuclear factor kappa-B; PAX8, paired box 8; T3, triiodothyronine; T4, thyroxine; D1, type I iodothyronine deiodinase; D2, type II iodothyronine deiodinase; Treg: regulatory T cells; Se+, selenium; SCFA, short-chain fatty acid.

However, relatively sparse research has been done on the possible link between the gut microbiota and thyroid disease, where the concept of ‘thyroid-gut-axis’ was proposed lately (11). One possible reason could be the spatially distant location of the thyroid gland in comparison to the gut, making the potential association between the two often negligible. However, few studies had been conducted in the last decade which investigated the thyroid-gut axis, owing to the advancement of microbial research and the development of microbiome assays. These studies indicated that the intestinal microbiota plays a pivotal role in thyroid disease pathogenesis and may act either by regulation of thyroid function through the uptake of thyroid-related micronutrients (12), metabolic enzymes derived from microbiota may regulate iodothyronine metabolism to affect thyroid hormone homeostasis (13), or may interact with the host immune cells and cytokines to regulate thyroid immunity (14, 15). This review thus summarizes the available literature related to the thyroid-gut axis, providing a theoretical foundation for future in-depth mechanistic studies, and a new perspective for realizing microecological treatment strategies for thyroid disease (**Table 1**, **Figure 1**).

**Table 1:** Overview of research on gut microbiota and thyroid diseases.

Diseases	Country	Year	Sample size	Methods	Ref
**TC**	China	2018	30 TC, 35 HC	16S rRNA gene sequencing, metabolomics	(16)
**TC & TN**	China	2018	20 TC, 18 TN, 36 HC	16S rRNA gene sequencing	(17)
**TC**	China	2022	90 TC, 90 HC	16S rRNA gene sequencing	(18)
**AITD (HT)**	China	2017	29 HT, 12 HC	DGGE, RT-PCR, Pyrosequencing	(19)
**AITD (HT)**	China	2018	28 HT, 16 HC	16S rRNA gene sequencing	(20)
**AITD (HT)**	China	2020	63 HT, 34 HC	16S rRNA gene sequencing	(21)
**AITD (HT and GD)**	Spain	2020	9 HT, 9 GD, 11 HC	16S rRNA gene sequencing	(22)
**AITD (HT and GD)**	Egypt	2021	7 HT, 13 GD, 30 HC	SYBR green RT-PCR	(23)
**AITD (hyperthyroidism)**	China	2014	14 hyperthyroidism, 7 HC	DGGE, RT-PCR, sequencing	(24)
**AITD (GD)**	China and Pakistan	2018	27 GD, 11 HC	DGGE, RT-PCR, 16S rRNA gene sequencing	(25)
**AITD (GD)**	China	2021	45 GD, 59 HC	16S rRNA gene sequencing	(26)
**AITD (GD)**	China	2021	15 GD, 14 HC	16S rRNA gene sequencing	(27)
**hypothyroidism**	China	2020	52 hypothyroidism, 40 HC	16S rRNA gene sequencing	(28)
**subclinical hypothyroidism**	China	2020	117 patients	16S rRNA gene sequencing	(29)

TC, thyroid cancer; HC, healthy controls; TN, thyroid nodule; AITD, autoimmunity thyroid diseases; HT, Hashimoto’s thyroiditis; GD, Graves’ Disease; RT-PCR, real time-polymerase chain reaction; DGGE, denaturing gradient gel electrophoresis.

## Thyroid homeostasis and intestinal microbiota

### Thyroid-related micronutrients and intestinal microbiota

The thyroid gland requires iodine as a complimentary micronutrient for the synthesis of thyroid hormones. Since the absorption through the gastrointestinal tract and transfer to the thyroid gland is the primary route of iodine uptake in humans, the trillions of microorganisms in the gut thus play a critical role in regulating iodine metabolism. Vought et al. investigated the relationships between total aerobic and anaerobic bacteria in the intestine of rats and the radioiodine uptake following kanamycin administration and revealed that both the bacterial enrichment *in vivo*, as well as the iodine uptake rate of the thyroid decreased, suggesting that the reduced intestinal microbiota may affect radioiodine uptake and thyroid function (30).

The iodine uptake is mainly achieved by the sodium/iodine symporter (NIS). It is speculated that altering the thyroid iodine metabolism by affecting the NIS expression and activity could be a potential pathway for lipopolysaccharides (LPS) and short-chain fatty acids (SCFAs) released by the gut microbiota (31, 32). The latest research supports the following critical mechanisms of thyroid homeostasis dysregulation caused by LPS. First, LPS directly affects thyroid cells by increasing the expression of thyroid-stimulating hormone (TSH)-stimulated thyroglobulin (Tg) and NIS genes, which was demonstrated in a rat thyroid cell line (FRTL-5) (31, 33). Second, LPS mediates downstream activation of nuclear factor kappa-B (NF-κB) through toll-like receptor 4 (TLR-4) on thyroid cells to regulate thyroid cell function (34). As another complementary pathway, the p65 subunit of NF-κB and paired box 8 (Pax8, the major regulator of NIS) can work together to promote NIS gene transcription (31). Notably, LPS being an endotoxin can inhibit the activity of hepatic type I iodothyronine deiodinase (D1) and conversely activates type II iodothyronine deiodinase (D2) in the hypothalamus and anterior pituitary, thus facilitating the conversion of thyroxine (T4) to triiodothyronine (T3) to affect thyroid function (35, 36). Similarly, SCFAs, an important metabolite of the intestinal microbiota, are also important in regulating NIS expression in thyroid cells. It is well established that SCFAs, especially butyric acid, can inhibit histone deacetylase (HDAC) and activate NIS re-expression in thyroid cancer cells, thereby inducing re-differentiation and iodine uptake (37, 38). This phenomenon, interestingly, was not limited to thyroid cancer cell lines, as consistent results were observed in other tumor cell lines such as MCF-7, Hep-G2, and MKN-7 (37, 38). Thus, it is envisaged that the sensitivity of tumor cells to radioactive iodine uptake could be increased by modulating the SCFAs to promote histone acetylation and enhance NIS expression, though further investigations are yet needed.

In addition to iodine, selenium is also detrimental to thyroid homeostasis, which is found in higher concentrations in the thyroid gland in comparison to any other tissue in the human body (39). Selenium, primarily selenoproteins such as glutathione peroxidase, deiodinase, etc. exerts its biological function in thyroid hormone metabolism, etc. (40). Selenium has been reported to promote the activity of CD4+CD25+FOXP3+ regulatory T cells (Treg), inhibit the secretion of cytokines, prevent apoptosis of follicular cells, and avert the secretion of cytokines in thyroiditis (39). The metabolism of selenium has been reported to be affected by the microbiota thus playing part in regulating thyroid functions possibly *via* an increased expression of Pax8 and promoting its binding to the upstream enhancer region of the NIS, which is assumed to be due to enhanced selenium uptake to activate NIS promoter and hence induce NIS transcription (41). Calomme et al. demonstrated that *Lactobacillus* converts intracellular sodium selenite into selenocysteine and selenomethionine, thus facilitating the absorption of selenium into the human body as organic selenium (42, 43).

Conversely, selenium has also been reported to alter the diversity and composition of gut microbiota in mice (44). Furthermore, microbiota may influence the absorption and utilization of microelements such as iron and zinc, which are used to regulate thyroid hormone synthesis or activity conversion (45). Fluctuations in the synthesis and metabolism of thyroid hormones targeting related microelements may result from microbial dysbiosis, leading to abnormal thyroid homeostasis (12).

### The role of microbiota in iodothyronine metabolism

It has been revealed that thyroid hormone homeostasis is closely related to the effect of gut microbiota on iodothyronine metabolism. On the one hand, the microbiota can promote its binding to iodothyronine and thus act as a reservoir. A study investigating this mechanism compared the ability of radioactive T3 and T4 binding in the cecum in normal and antibiotic-treated rats, where a significant decrease in binding ability of T3 and T4 was observed in comparison to normal rats (46). It is worth mentioning that this role of microbiota has a positive effect in maintaining thyroid hormone homeostasis and effectively reduces the need for levothyroxine (L-T4). Spaggiari et al. evaluated the impact of a ‘probiotic’ mixture (*Lactobacillus* and *Bifidobacterium*) on L-T4 in patients with hypothyroidism, where the ‘probiotic’ mixture was found to significantly reduce the requirement for L-T4 thereby helping in the prevention of serum thyroid hormone fluctuations (47). Similar results were also reported by Yao et al., where the dose of L-T4 required to maintain the stable TSH level was found related to the microbiota (29).

Furthermore, the microbiota can uncouple the sulfated glucuronide derivatives of iodothyronine *via* bacterial sulfate esterase or β-glucuronidase, thereby improving the reabsorption of thyroid hormones in the enterohepatic circulation (13, 48). In addition, inhibition of 5- deiodinase activity by microbiota is another pathway for its involvement in iodothyronine metabolism, the direct effect of which is translated in a reduction in the conversion of T4 to T3 and rT3. Deiodinase activity in the adult rat intestine was found significantly lower than in the rat fetus, which could be attributed to the inhibition of resident intestinal microflora (49). It is hence concluded that the microbiota is associated with iodothyronine metabolism through a complex thyroid-gut axis regulatory pathway. However, refined mechanistic studies are required to clarify the role of intestinal microbiota.

### Thyroid autoimmunity and intestinal microbiota

As one of the most common organ-specific autoimmune diseases, autoimmune thyroid diseases (AITD) are characterized by the presence of thyrotrophin receptor antibody (TRAb), thyroid peroxidases antibody (TPOAb), and thyroglobulin antibody (TGAb) against thyroid cells resulting in Hashimoto’s thyroiditis (HT) and Graves’ disease (GD) (50, 51). At present, AITD pathogenesis remains not fully understood. It is generally caused by the interaction between various endogenous and exogenous factors such as genetic susceptibility, environmental factors, and immune disorders (52, 53). Although there is no experimental evidence elucidating the mechanism of microbiota and thyroid autoimmunity, related studies suggest that microbiota and its metabolites may directly or indirectly modulate thyroid immunity, thereby inducing AITD (54, 55). Generally, one of the mechanisms of microbiota-associated AITD is antigenic cross-reactivity between gut microbiota and thyroid, where one is reported to be due to cross-immune response induced by *Yersinia enterocolitica via* thyrotropin receptor (TSHR)-like substances, while another by the *Bifidobacterium* and *Lactobacillus via* TPO Tg-like substances (56, 57). A high prevalence of cytotoxin-associated gene A (Cag-A) antigen-positive *Helicobacter pylori* (Hp) infection has also been discovered in GD patients, which has been attributed to the similarity of Cag-A positive Hp strains to TPO sequences (58).

Additionally, the microbiota-derived SCFAs can be involved in the pathogenesis and progression of AITD through immune regulation by T helper 17 (Th17)/Treg and cytokines (14). As prompted by the previous, the microbiota and its metabolites breaking the intestinal barrier reaching the systemic circulation and promoting the release of inflammatory factors may also be one of the mechanisms of inflammatory activation in AITD (7, 59). In conclusion, the correlation between microbial dysbiosis and AITD remains relatively poor. More research on how microbiota regulates thyroid immunity has to be done.

## Benign thyroid disease and intestinal microbiota

### The association between HT and microbiota

In 2012, Kouki Mori et al. proposed that commensal microbiota may be an important environmental factor in triggering HT [56]. Even though this opinion was based on indirect research evidence, it was influential in promoting subsequent research. Since 2017, there has been a spike in research on gut microbiota and thyroid diseases globally, particularly in AITD. Ishaq et al. demonstrated the correlation between the altered composition and increased diversity of the microbiota in HT patients relative to healthy individuals, where the microbiota of HT patients was found impaired (60). Similar results were reported by Zhao et al. stating that the changes in the gut microbes are correlated with the thyroid function (61). It is important to note that both of the above studies found a declined abundance of *Prevotella* in HT patients, which is consistent with that found in MS and hepatocellular carcinoma (16, 17). It has been established that *Prevotella* is correlated with reduced pro-inflammatory Th17 polarization, anti-inflammatory Treg differentiation, and the production of anti-inflammatory metabolites in the gut (62). Therefore, it is speculated that the Th17/Treg homeostasis regulation might be a potential pathogenic pathway for *Prevotella* in HT patients. In contrast, Liu et al. collected fecal samples from HT patients in different stages of thyroid function (45 HT patients with euthyroidism, 18 HT patients with hypothyroidism) to further explore the gut microbiota characteristics in HT patients and revealed that the microbial abundance and diversity in HT patients were significantly lower than those of normal patients, where the Lachnospiraceae incertae sedis, *Lactonifactor*, *Alistipes*, and *Subdoligranulum* were enriched considerably in HT patients in comparison to euthyroidism (63). Although the results of the above studies differed, it is believed to be reasonable and may be due to the interference caused by environmental factors such as assay technique, the subject’s diet, and lifestyle on the intestinal microecology.

Two small sample studies have recently conducted a preliminary investigation of comparing the microbiota between AITD patients (HT and GD) and healthy controls (64, 65). Isabel et al. stated that different types of AITD had specific alterations in microbiota, where microbial abundance was found increased in HT, and microbial evenness was reduced in GD compared to healthy controls, suggesting that the altered microbiota might be related to the development of the immune system and intolerance to autoantigens in AITD (64). Similar abnormalities in the microbial structure in patients with AITD were also discovered by Hanaa et al. (65). Although the associations of HT patients with gut microbiota are now well established, the causal relationships between microbiota and HT and the specific mechanisms remain unknown. Large sample size studies and more rigorous mechanistic experiments remain necessary.

### The association between GD and microbiota

The population of *Bifidobacterium* and *Lactobacillus* tend to reduce with a significant increase in *Enterococcus* content in hyperthyroid patients following quantification of some bacterial genera using real-time PCR (18). Generally, the *Bifidobacterium* and *Lactobacillus* are considered beneficial bacteria that help maintain host health through mechanisms like immune regulation and intestinal permeability. Hence, both the *Bifidobacterium* and *Lactobacillus* are considered probiotics, and their administration in mice was found to prevent the development of autoimmune diseases including type 1 diabetes and colitis (66). Conversely, Ishaq HM et al. also observed dysbiosis of the intestinal microbiota in GD patients (19). It is worth mentioning that *Lactobacillus* appears to be a double-edged sword. Studies have demonstrated that specific strains of *Bifidobacterium* and *Lactobacillus* are pathogenic due to structural homology with the amino acid sequences of human TPO and Tg and thus can induce AITD through a cross-antigen-molecular mimicry mechanism (57). Our previous study analyzing the fecal microorganisms of 45 GD patients and 59 healthy controls using 16S rRNA sequencing revealed that the abundance of *Lactobacillus* was significantly higher in TPOAb-positive GD patients than that in TPOAb-negative GD patients, thus suggesting that *Lactobacillus* may play a vital role in AITD pathogenesis (20). Our findings were echoed in another clinical study, in which *Lactobacillus* enriched in GD patients was found to be associated with thyroid autoimmune antibodies (67). It is evident that there is no absolute “good” or “bad” intestinal microbiota, and the complex mechanisms of intestinal microbiota in GD must be further explored in a targeted manner.

Studies on intestinal microbiota in animal models of GD conducted in recent years have indicated that intestinal flora may influence the construction of mouse GD models. The incidence of hyperthyroidism and orbital tissue changes were reported to be less severe in C57BL/6 J mice than those in BALB/c mice GD models, in addition to microbial structural alterations found different in C57BL/6 J from that of BALB/c mice (68). Similarly, in another study, BALB/c mice receiving the same immunization methods in other experimental centers showed different effects of GD phenotype (69). The differences in microbiota and disease phenotypes observed in these various studies support an important role for gut microbiota in modulating the thyroid immune response. Furthermore, Moshkelgosha et al. discovered that abnormal intestinal microbiota exacerbated the development of GD as an important pathogenic factor through fecal microbiota transplantation experiments (21). These studies reveal that microbiota plays an essential role in TSHR-induced GD pathogenesis and that microbiota may contribute to the heterogeneity of the induction response.

### The association between hypothyroidism and microbiota

It has been previously suggested that hypothyroidism is often accompanied by impaired gastrointestinal motility, which creates favorable conditions for colonization and overgrowth of intestinal bacteria (22). Overgrown bacteria, in turn, promote the impaired neuromuscular function of the gastrointestinal tract, further exacerbating chronic gastrointestinal symptoms in hypothyroid patients (22). Studies have confirmed that excessive growth of tiny intestinal bacteria occurred in hypothyroid patients, which were found to be inhibited with improvement in gastrointestinal symptoms following antibiotic therapy (23, 24). Therefore, hypothyroidism and microbiota are considered in a reciprocal relationship and that assessing the growth of tiny intestinal bacteria could be instructive in managing hypothyroid patients.

Recently, the intestinal microbiota and thyroid metabolism in hypothyroid patients have also been studied. As previously mentioned, *Lactobacillus* and *Bifidobacterium* mixtures did not directly alter the thyroid function compensation in hypothyroid patients but helped prevent fluctuations in serum thyroid hormones (47). A clinical trial found that synbiotics may have beneficial effects on thyroid function in hypothyroid patients. However, the difference was not statistically significant in comparison to the placebo group (70). Furthermore, it has been observed that the microbial composition and function were altered in primary hypothyroidism patients. *Veillonella*, *Paraprevotella*, *Neisseria*, and *Rheinheimera* can distinguish primary hypothyroidism patients from healthy individuals (25). It is noteworthy that reduced levels of SCFAs, caused by a decrease in SCFA-producing bacteria in the gut of primary hypothyroidism patients, exacerbate the intestinal barrier’s impairment and elevated serum lipopolysaccharide levels. The authors also confirmed that altered intestinal microbiota promoted hypothyroidism in mice by fecal microbiota transplantation. This is the first report to provide a comprehensive and rigorous demonstration of the causal role of the thyroid and the intestine through clinical cross-sectional studies and animal studies. Subsequently, a cross-sectional study analyzed the microbiota in patients with mild subclinical hypothyroidism treated with or without L-T4, where the dose of L-T4 required to maintain TSH levels and the progression of subclinical hypothyroidism was found associated with gut microbes, which is probably a result of the microbiota influencing L-T4 metabolism (29). These studies strongly supported the correlations between primary hypothyroidism and microbial dysbiosis and are of great significance for subsequent studies on the mechanisms of gut-thyroid interactions.

## The association between thyroid cancer and microbiota

The microbiota and its metabolites are important endogenous factors that can influence the development of various cancers (26). However, few studies on gut microbiota in thyroid cancer patients have recently been published. A serum metabolomic analysis of patients with distant metastases from thyroid cancer revealed that diet and gut microbiota interactions might play an essential role in tumor aggressiveness (27). Subsequently, a small sample study conducted by Feng et al. integrated 16S rRNA gene sequencing and non-target metabolomics, and the results revealed that the microbiota of the thyroid cancer group had higher abundance and diversity than that of healthy controls and that alterations were strongly associated with serum lipid metabolites (71). Furthermore, a practical model consisting of eight metabolites combined with five genera was constructed based on multi-omics data to characterize the intestinal microecology of these thyroid cancer patients. Zhang et al. investigated the correlation between thyroid cancer, thyroid nodules, and microbiota by comparing the microbial structure characteristics between 20 thyroid cancer patients, 18 thyroid nodules patients, and 36 healthy controls utilizing high-throughput sequencing, and results indicated that the thyroid cancer and thyroid nodules were closely associated with altered microbiota (72). It may be noted that both studies found a reduced abundance of SCFAs-producing bacteria (e.g., *Lachnospiraceae*, and *Butyricimonas*) in patients with thyroid cancer. SCFAs are thought to have a regulatory effect on the immune microenvironment, where their reduced levels may also lead to increased cell death and cell renewal, causing an increased risk of cancer (73, 74). An advanced study of the mechanisms of intestinal microbiota and SCFAs in thyroid cancer will therefore contribute to our further understanding of the pathophysiology of thyroid cancer. A case report published recently observed a significant increase in the microbiota alpha diversity in patients with undifferentiated thyroid cancer treated with the anti-PD-1 monoclonal antibody; Pembrolizumab (75). Another short review suggested that microbiota may influence radioiodine refractory papillary thyroid cancer by modulating different mechanisms associated with NIS (76).

In contrast to previous studies, our new cross-sectional survey of a larger sample identified potential microbiota associated with tumor lymph node metastasis, including *Fusobacterium*, *Alistipes*, *Hungatella*, and *Phascolarctobacterium*, in addition to dysbiosis of the gut microbiota in patients with thyroid cancer. The pathogenicity of these genera has been demonstrated in several diseases, and we have accordingly inferred that thyroid cancer progression may be linked to intestinal microbiota affecting circulating DNA methyltransferase levels (28). The research found a strong link between altered intestinal microbiota and thyroid cancer. However, the presumptions are yet in their early stages. The causal relationships and mechanisms of interaction between the two still need to be further clarified by large samples, multi-omics studies, and *in vitro* and *in vivo* experiments.

## Conclusions

As research into the gut microecology of thyroid diseases progresses, there is increasing evidence that gut microbiota is an important environmental factor directly or indirectly influencing the progression of thyroid diseases and that thyroid diseases can exacerbate disturbances in the microbiota. Inducing immune-inflammatory responses, altering iodothyronine metabolism, and affecting thyroid-associated micronutrient absorption are potential pathways through which the microbiota and metabolites are involved in thyroid homeostasis. Nevertheless, the causal relationships between gut microbiota and thyroid diseases, how gut microbiota modulates thyroid autoimmunity, and the specific mechanisms by which a particular bacterium or core flora triggers thyroid diseases, are still poorly understood. Therefore, there is still a great deal of evidence to be sought through multi-omics integration analysis, cellular and animal experiments. It is believed that as research progresses, future scientific advances in the study of thyroid-gut axis will be advanced, leading to a more defined relationships and mechanisms as well as gut microecological therapies.

## Author contributions

WJ prepared the draft manuscript under the guidance and supervision of DL, ZL, GL, and DG. The manuscript was substantively revised by DL and ZL. All authors contributed to the article and approved the submitted version.

## Funding

This study was sponsored by National Natural Science Foundation of China [82071964], Shanghai Leading Talent Program [03.05.19005], Shanghai Shenkang Three-year Action Project [SHDC2020CR2054B], and Shanghai Municipal Health Commission [GWV-10.1-XK9], and Natural Science Foundation of Shanghai [21ZR1449600].

## Conflict of interest

The authors declare that the research was conducted in the absence of any commercial or financial relationships that could be construed as a potential conflict of interest.

## Publisher’s note

All claims expressed in this article are solely those of the authors and do not necessarily represent those of their affiliated organizations, or those of the publisher, the editors and the reviewers. Any product that may be evaluated in this article, or claim that may be made by its manufacturer, is not guaranteed or endorsed by the publisher.
